# OR2-002 – The risk of FMF in MEFV heterozygotes

**DOI:** 10.1186/1546-0096-11-S1-A2

**Published:** 2013-11-08

**Authors:** I Jéru, V Hentgen, E Cochet, P Duquesnoy, G Le Borgne, E Grimprel, K Stankovic Stojanovic, S Karabina, G Grateau, S Amselem

**Affiliations:** 1 UMR_S933, INSERM, France; 2 Université Pierre et Marie Curie-Paris6, France; 3 Service de Génétique, APHP, Hôpital Trousseau, Paris, France; 4 Centre de Référence des Maladies AutoInflammatoires, Centre Hospitalier de Versailles, Le Chesnay, France; 5 Service de Pédiatrie Générale, APHP, Hôpital Trousseau, France; 6 Service de Médecine Interne, APHP, Hôpital Tenon, Paris, France

## Introduction

Familial Mediterranean fever (FMF) is an autosomal recessive autoinflammatory disorder due to *MEFV* mutations and one of the most frequent Mediterranean genetic diseases. The observation of many heterozygous patients in whom a second mutated allele was excluded led to propose that heterozygosity could be causal; however, this might often be coincidental due to the very high rate of mutations in Mediterranean populations.

## Objectives

To better delineate the pathogenicity of heterozygosity in order to help genetic counselling and better manage the disease.

## Methods

Complementary statistical approaches were used: estimation of FMF prevalence at population levels, genotype comparison in siblings from 63 familial forms, and genotype study in 557 patients from four Mediterranean populations.

## Results

At population level, we did not observe any contribution of heterozygosity to the disease prevalence. In affected siblings of patients carrying two *MEFV* mutations, 92% carry two mutated alleles whereas 4% are heterozygous with typical FMF diagnosis. We also demonstrated statistically that patients are more prone to be heterozygous than healthy individuals, as shown by the higher ratio heterozygous carriers/non carriers in patients (p<10^-7^ - p<0.003). The risk for heterozygotes to develop FMF was estimated between 2.1x10^-3^ and 5.8x10^-3^ and the relative risk, as compared to individuals carrying no *MEFV* mutation, between 6.3 and 8.1.

## Conclusion

This is the first statistical demonstration that heterozygosity is not responsible for classical Mendelian FMF, but constitutes a susceptibility factor for clinically-similar complex conditions. We also provide a first estimate of the risk for heterozygotes to develop FMF.

## Disclosure of interest

None declared.

